# Prevalence of rheumatoid factor in asymptomatic chronic hepatitis B surface carriers in the context of endemic malaria: cross-sectional study at University Hospital of Cocody, Abidjan, Côte d'Ivoire

**DOI:** 10.11604/pamj.2025.50.111.46406

**Published:** 2025-04-24

**Authors:** Amah Patricia Victorine Goran-Kouacou, Oppong Richard Yéboah, Aya Ursule Aniela Assi, Yida Jocelyne Séri, Adjoumanvoulé Honoré Adou, Koffi N´Guessan, Séry Romuald Dassé

**Affiliations:** 1 Laboratory of Immunology and Haematology, University Hospital of Cocody, Abidjan, Côte d'Ivoire,; 2 Department of Immunology-Allergology, Medical Sciences Training and Research Unit, Félix Houphouët-Boigny University, Abidjan, Côte d'Ivoire

**Keywords:** Rheumatoid factor, chronic hepatitis B, autoimmunity, antigenic stimulation, immunosenescence, Côte d’Ivoire

## Abstract

**Introduction:**

rheumatoid factor is an autoantibody involved in autoimmune diseases and certain chronic infections. Chronic hepatitis B, common in Côte d'Ivoire, leads to prolonged antigenic stimulation and chronic inflammation, which may promote rheumatoid factor production. This study aimed to determine the prevalence of rheumatoid factor and to identify factors associated with rheumatoid factor positivity in asymptomatic chronic carriers of hepatitis B surface antigen in a hospital setting in Abidjan, Côte d´Ivoire.

**Methods:**

a prospective cross-sectional study was conducted in 2024 at the University Hospital of Cocody in Abidjan, Côte d'Ivoire. A total of 120 asymptomatic chronic carriers of hepatitis B surface antigen without autoimmune diseases or active malaria were enrolled. Clinical and demographic data were collected. Rheumatoid factor was measured by immunoturbidimetry with a positivity threshold of >14 IU/mL. Multivariable logistic regression analysis was used to identify factors associated with rheumatoid factor positivity.

**Results:**

the prevalence of rheumatoid factor positivity was 12.5% (95% CI: 6.54-18.46). The mean age of participants was 36.4 ± 11.2 years, and 60% were male. Advanced age (≥30 years) was independently associated with rheumatoid factor positivity (aOR: 3.32; 95% CI: 1.06-10.39; p = 0.04). No significant association was found with sex or reason for consultation.

**Conclusion:**

in this hospital-based population of asymptomatic chronic carriers of hepatitis B surface antigen, advanced age was associated with rheumatoid factor positivity. Monitoring rheumatoid factor in older carriers may help improve the assessment of autoimmune risks and diagnostic accuracy.

## Introduction

Rheumatoid factor (RF) is a heterogeneous family of autoantibodies directed against the crystallizable fragment (Fc) of immunoglobulin G (IgG). Primarily of the immunoglobulin M (IgM) isotype, it may also include other classes such as immunoglobulin A (IgA) and IgG [1]. These antibodies can be divided into two categories according to their affinity and function. Physiological RFs, polyreactive and of low affinity, contribute to immune regulation by facilitating the clearance of immune complexes and contributing to anti-infectious defense mechanisms [2]. Pathological RFs, monoreactive and of high affinity, are associated with various pathological conditions [2]. Initially recognized as a diagnostic marker of rheumatoid arthritis [3,4], RF is also detected in a range of diseases, including autoimmune diseases, cancers, and chronic infections [4,5]. Chronic hepatitis B, caused by the hepatitis B virus (HBV), is one such infection. It represents a major public health problem, particularly in Côte d'Ivoire, where the prevalence of hepatitis B surface antigen (HBsAg) carriage exceeds 8% [6]. This infection induces prolonged antigenic stimulation and chronic inflammation, two mechanisms known to promote the production of autoantibodies such as RF [5]. However, the relationship between HBV infection and RF production remains poorly understood. In addition, pathological RF can interfere with diagnostic tests by binding to immune complexes or through cross-reactivity with antigens, complicating serological test interpretation [7,8]. This is of particular concern in settings with limited medical resources, where diagnostic accuracy is critical to ensure appropriate patient management. Understanding the relationship between chronic HBV infection and RF production is essential to improve the interpretation of serological tests and to identify the immunopathological risks associated with this chronic viral infection. This study aimed to determine the prevalence of RF in asymptomatic chronic HBsAg carriers followed at the University Hospital of Cocody in Abidjan, Côte d'Ivoire, and to identify the clinical and immunological factors associated with RF positivity.

## Methods

**Study design and setting:** this was a prospective, descriptive and analytical cross-sectional study conducted over a 12-month period, from January to December 2024, at the Immunology and Haematology Laboratory of the University Hospital of Cocody, a tertiary referral center located in the commune of Cocody, Abidjan, Côte d'Ivoire. This hospital is affiliated with Félix Houphouët-Boigny University and is a center of excellence for the management of infectious, hepatic, and autoimmune diseases. The Immunology and Hematology Laboratory performs immunological, hematological, and biochemical investigations as part of diagnostic and follow-up care.

**Study population:** the study population consisted of asymptomatic chronic HBsAg carriers who were either being followed up or presenting for initial screening at the University Hospital of Cocody during the study period.

**Inclusion criteria:** i) age 15 years and older. ii) Resident in Abidjan for at least one year. iii) Confirmed chronic HBsAg carrier (persistent positivity after six months for initial screening cases). iv) Normal transaminase levels (ALT/AST). v) Negative HBeAg and positive anti-HBe antibodies. vi) Negative anti-HBc IgM antibodies. vii) No active malaria (absence of fever and negative smear).

**Exclusion criteria:** known autoimmune disease, ongoing antiviral or immunosuppressive treatment, acute infection at the time of enrolment, indeterminate serological results for HBsAg, HBeAg or rheumatoid factor.

**Sample size:** the minimum sample size was calculated assuming an expected prevalence of rheumatoid factor of 7%, as reported by Sombo *et al*. [9] in healthy individuals in Côte d'Ivoire. The formula used was:


$$n=\frac{Z^{2}\times p\left(1-p\right)}{d^{2}}$$

where: n= sample size, Z= critical value for a 95% confidence interval (1.96), p= expected prevalence (0.07), d= margin of error (0.05). After adjusting for a 10% non-response rate, the minimum sample size required was 110 patients. Finally, 120 patients were included to ensure adequate statistical power.

**Sampling method:** convenience sampling was used. All patients who met the inclusion criteria and presented during the study period were recruited.

**Data collection:** data were collected using a structured questionnaire that included the following information: socio-demographic information (age, gender); medical history (transfusions, infections, treatments); reason for consultation (initial screening or chronic follow-up). Blood samples were taken from each patient as follows: i) Dry tube: for measurement of HBsAg, transaminases (ALT/AST), HBeAg, anti-HBe antibodies, anti-HBc IgM and rheumatoid factor. ii) Ethylenediaminetetraacetic acid (EDTA) tube: for the preparation of a Giemsa-stained thick blood smear to exclude active malaria. Patients recruited at baseline were followed up after six months to confirm their chronic HBsAg carrier status.

**Laboratory analysis:** virological, biochemical and immunological tests were performed using the Cobas 6000 analyser (Roche Diagnostics), according to the following principles, as summarized in Table 1. i) HBsAg, HBeAg, anti-HBe antibodies, anti-HBc IgM: measured by electrochemiluminescence immunoassay (ECLIA) on the e601 module. ii) Transaminases (ALT/AST): measured by spectrophotometric method on the c501 chemistry module. iii) Rheumatoid factor: measured by latex immunoturbidimetry on the c501 chemistry module, with a positivity threshold set at > 14 IU/mL. Internal and external quality controls were carried out to ensure the reliability of the results.

**Table 1:** summary of viral markers

Clinical Status	HBs Ag	IgM anti-HBc	HBe Ag	Anti-HBe Ab
Acute infection	Positive	Positive	Variable	Negative
Chronic replicative infection	Positive	Negative	Positive	Negative
Chronic inactive infection	Positive	Negative	Negative	Positive

HBsAg: Hepatitis B surface antigen; IgM anti-HBc: Immunoglobulin M antibody to hepatitis B core antigen; HBeAg: Hepatitis B e antigen; Anti-HBe Ab: Antibody to hepatitis B e antigen

**Variables studied:** rheumatoid factor; age (divided into age categories); sex; reason for consultation (screening or follow-up).

**Data analysis:** data were entered and analysed using Epi Info software (version 3.5.1): i) Qualitative variables were expressed as frequencies and percentages. ii) Quantitative variables were expressed as mean ± standard deviation. Univariable analysis was performed using the chi-squared test, with a significance threshold of p < 0.05. iii) Multivariable logistic regression analysis was performed to identify factors independently associated with rheumatoid factor positivity. Results were expressed as adjusted odds ratios (aOR) with 95% confidence intervals and p values. Incomplete data for any variable of interest led to the exclusion of the participant from the specific analysis.

**Ethical considerations:** the study was conducted in accordance with the tenets of the Declaration of Helsinki. Ethical approval was obtained from the Ethics Committee of the University Hospital of Cocody, Abidjan, Côte d´Ivoire (Ref: 010/MSHP-CMU/CHU-C/DMS/RK/25). Written informed consent was obtained from all participants before enrolment. Confidentiality and anonymity of data were ensured throughout the study.

## Results

**Figure 1:**
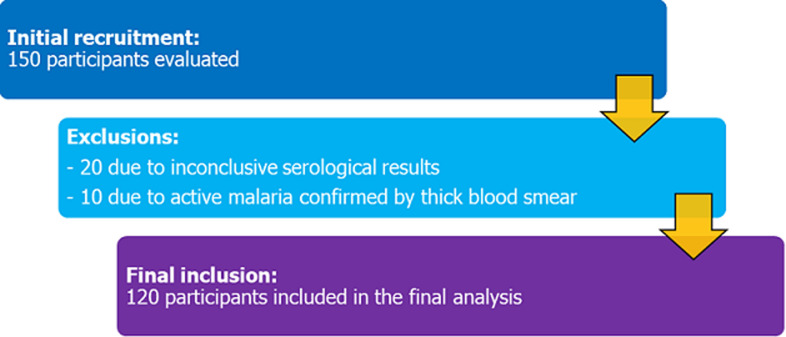
flow diagram of study participants

**Participants and selection:** a total of 150 participants were initially recruited. After applying the inclusion and exclusion criteria, 120 participants were retained for the final analysis, exceeding the minimum required size of 110 to ensure adequate statistical power (Figure 1).

**Characteristics of patients:** the characteristics of the study population are summarized in Table 2. Among the 120 participants, 72 (60.0%) were male and 48 (40.0%) were female, with a sex ratio of 1.5 in favor of male patients. The mean age was 36.4 ± 11.2 years (range: 16 to 65 years). Patients aged 30 years or older represented 66.6% of the study population (n= 80). Regarding the reason for consultation, 75 patients (62.5%) were recruited during initial HBsAg screening, while 45 (37.5%) were under chronic follow-up.

**Table 2:** socio-demographic characteristics of patients studied

Characteristics	Number (n=120)	Frequency (%)
**Sex**		
Male	72	60.0
Female	48	40.0
**Age (years)**		
16-29	40	33.3
30-50	55	45.8
51-65	25	20.8
**Mean age**	36.4 ± 11.2	
Range	16 - 65	
**Reason for consultation**		
Initial screening	75	62.5
Chronic follow-up	45	37.5

**Prevalence of rheumatoid factor and factors associated with rheumatoid factor positivity:** the rheumatoid factor was detected in 15 participants, representing a prevalence of 12.5% (95% CI: 6.54-18.46). The results of the univariable and multivariable logistic regression analyses are summarized in Table 3. Advanced age (≥30 years) was the only factor independently associated with RF positivity (aOR: 3.32; 95% CI: 1.06-10.39; p= 0.04). Sex and reason for consultation were not significantly associated with RF positivity: male sex (aOR: 1.39; 95% CI: 0.45-4.27; p= 0.768), chronic follow-up (aOR: 1.13; 95% CI: 0.38-3.39; p= 0.799).

**Table 3:** univariable and multivariable analysis of factors associated with rheumatoid factor positivity

Variables	Total (n = 120)	RF positive cases (n= 15)	Univariable OR (95% CI)	P-value	Multivariable aOR (95% CI)	P-value
**Sex**						
Male	72	10	1.39 (0.45-4.27)	0.768	-	-
Female	48	5	Ref		Ref	
**Age (years)**						
≥ 30	80	12	3.32 (0.90-12.21)	0.022	3.32 (1.06-10.39)	0.04
< 30	40	3	Ref		Ref	
**Reason for consultation**						
Chronic follow-up	45	6	1.13 (0.38-3.39)	0.799	-	-
Initial screening	75	9	Ref		Ref	

RF: Rheumatoid factor; OR: Odds ratio; aOR: Adjusted odds ratio; CI: Confidence interval; Ref: Reference category. Significant p-value < 0.05

## Discussion

This study aimed to determine the prevalence of RF in asymptomatic chronic HBsAg carriers followed at the University Hospital of Cocody in Abidjan, Côte d´Ivoire, and to identify factors associated with RF positivity. The prevalence of RF was 12.5% (95% CI: 6.54-18.46), higher than the 7% prevalence reported in healthy individuals in Côte d´Ivoire [9]. Advanced age (≥30 years) was the only factor independently associated with RF positivity (aOR: 3.32; 95% CI: 1.06-10.39; p = 0.04). No association was found with sex or reason for consultation. Our findings support the hypothesis that chronic inflammation and prolonged antigenic exposure due to HBV infection promote RF production through multiple immunopathological mechanisms: i) Persistent antigenic stimulation: prolonged exposure to HBsAg leads to sustained activation of antigen-presenting cells (APCs), CD4+ T cells, and B cells, driven by pro-inflammatory cytokines (IL-4, IL-6, TNF-α) [10]. ii) Disruption of immune tolerance: chronic inflammation impairs regulatory T cell function, allowing autoreactive B-cell clones to expand [11]. iii) Epitope spreading and neoantigen formation: chronic HBV infection can expose cryptic self-antigens, generate neoantigens, and induce stress-related protein modifications, triggering autoantibody production [12-14]. iv) Immune complex formation: rheumatoid factor binds to IgG, forming immune complexes that activate the complement system, further amplifying chronic inflammation [15,16].

The association between advanced age and RF positivity can be explained by immunosenescence, a well-characterized process involving thymic involution, reduction in naïve T cells, dysfunction of regulatory T cells, and chronic low-grade inflammatory state (inflammaging), driven by the pro-inflammatory senescence-associated secretory phenotype (SASP) phenotype [17-19]. Cumulative antigenic exposure throughout life, particularly in chronic HBsAg carriers, may further exacerbate these immune alterations [10]. In our study, RF prevalence increased from 5% in participants under 30 years to 16% in those over 50 years, reinforcing this age-related immune dysregulation hypothesis. Interestingly, no association was found with female sex, a well-known risk factor for autoimmune diseases. Although hormonal and genetic factors contribute to increased autoimmune susceptibility in women [20,21], the chronic antigenic stimulation induced by HBV may override this sex-based predisposition in this specific population. Similarly, the absence of association with reason for consultation suggests that RF production is not necessarily influenced by the duration of HBV carriage. However, the limited number of RF-positive cases in the chronic follow-up group (6 out of 45) reduces the statistical power to fully explore this relationship. These findings have two main clinical implications: i) Rheumatoid factor screening in older HBsAg carriers may help identify patients at risk of autoimmune complications. ii) Pathological RF can interfere with serological tests, leading to diagnostic errors, particularly in resource-limited settings [7,8].

**Strengths and limitations of the study:** this is the first study in Côte d´Ivoire to assess RF prevalence in chronic HBsAg carriers, providing original data on autoantibody production in this context. However, some limitations must be considered: i) Cross-sectional design: this study does not allow causal inference regarding the relationship between HBV infection and RF production. ii) Recalling bias: self-reported medical history may have been affected by recall bias, despite efforts to ensure data reliability. iii) Lack of HBV viral load and inflammatory markers: the absence of virological and immunological markers prevents a more detailed exploration of HBV replication status and immune activation as potential contributors to RF production. Further longitudinal studies incorporating HBV viral load, liver enzymes levels, and inflammatory cytokines are needed to better understand the evolution of autoantibody responses in chronic HBsAg carriers. While these findings are particularly relevant in a hospital-based setting in Abidjan, their generalizability to other populations requires caution, considering potential environmental and demographic differences.

## Conclusion

This study highlights an increased prevalence of rheumatoid factor in asymptomatic chronic HBsAg carriers followed at the University Hospital of Cocody, Abidjan, Côte d´Ivoire. Advanced age was the main factor associated with RF positivity. These findings underscore the importance of immunological monitoring, particularly in older HBsAg carriers, to improve the assessment of autoimmune risks and enhance the diagnostic reliability of serological tests in this context.

### What is known about this topic

- Rheumatoid factor is an autoantibody classically associated with autoimmune diseases, but it can also be detected in certain chronic infections; however, its association with chronic hepatitis B remains poorly studied in our context;
- In the context of chronic hepatitis B surface antigen carriers monitored in a hospital setting in Abidjan, repeated infectious episodes could potentially exacerbate antigenic stimulation and contribute to rheumatoid factor production.

### What this study adds

- This study reveals a prevalence of rheumatoid factor of 12.5% in asymptomatic chronic hepatitis B surface antigen carriers followed at the University Hospital of Cocody in Abidjan, Côte d´Ivoire;
- It also highlights a significant association between advanced age and rheumatoid factor positivity, suggesting the role of immunosenescence and prolonged antigenic exposure in this population;
- Our findings provide novel data on rheumatoid factor production in chronic hepatitis B surface antigen carriers in this specific hospital setting, offering new perspectives for improving the monitoring and understanding of autoimmune mechanisms associated with HBV infection.

## References

[1] Sieghart D, Platzer A, Studenic P, Alasti F, Grundhuber M, Swiniarski S (2018). Determination of autoantibody isotypes increases the sensitivity of serodiagnostics in rheumatoid arthritis. Front Immunol.

[2] Eurofins Biomnis Rheumatoid Factors.

[3] Maibom-Thomsen SL, Trier NH, Holm BE, Hansen KB, Rasmussen MI, Chailyan A (2019). Immunoglobulin G structure and rheumatoid factor epitopes. PLoS One.

[4] Guliaev SV, Strizhakov LA, Moiseev SV (2023). Rheumatoid factor: study history and concept evolution. Ter Arkh.

[5] Moll J, Isailovic N, De Santis M, Selmi C (2019). Rheumatoid factors in Hepatitis B and C infections: connecting viruses, autoimmunity, and cancer. Isr Med Assoc J.

[6] World Health Organization Regional Office for Africa. 91 Million Africans Infected with Hepatitis B or C.

[7] N'Guessan K, Dassé SR, Yéboah OR, Kouacou AP, Séka SJ (2014). Interférences hétérophiles : expérimentation d'une méthode d'épuisement des sérums en facteurs rhumatoïdes à Abidjan (Côte d'Ivoire). Med Sante Trop.

[8] Lee JH, Jang JW, Cho CH, Kim JY, Han ET, Yun SG (2014). False-positive results for rapid diagnostic tests for malaria in patients with rheumatoid factor. J Clin Microbiol.

[9] Sombo MF, Dasse SR, Akre DP, N'guessan K, Sangare MA (2006). Fréquence du facteur rhumatoïde chez les sujets sains vivant dans une zone d'endémie palustre. Rev Int Sci Méd.

[10] Rosenblum MD, Remedios KA, Abbas AK (2015). Mechanisms of human autoimmunity. J Clin Invest.

[11] Haskins K, Buckner JH (2016). Editorial overview: Autoimmunity. Curr Opin Immunol.

[12] Dubucquoi Sylvain Polyarthrite rhumatoïde: auto-antigènes et auto-anticorps.

[13] Vallée D, Blanc M, Lebeaupin C, Bailly-Maitre B (2020). La réponse au stress du réticulum endoplasmique dans la physiopathologie des maladies chroniques du foie. Med Sci (Paris).

[14] Mustelin T, Andrade F (2024). Autoimmunity: the neoantigen hypothesis. Front Immunol.

[15] Coss SL, Zhou D, Chua GT, Aziz RA, Hoffman RP, Wu YL (2023). The complement system and human autoimmune diseases. J Autoimmun.

[16] Lintner KE, Wu YL, Yang Y, Spencer CH, Hauptmann G, Hebert LA (2016). Early Components of the Complement Classical Activation Pathway in Human Systemic Autoimmune Diseases. Front Immunol.

[17] Liu Z, Liang Q, Ren Y, Guo C, Ge X, Wang L (2023). Immunosenescence: molecular mechanisms and diseases. Signal Transduct Target Ther.

[18] Thomas R, Wang W, Su DM (2020). Contributions of Age-Related Thymic Involution to Immunosenescence and Inflammaging. Immun Ageing.

[19] Wang Y, Dong C, Han Y, Gu Z, Sun C (2022). Immunosenescence, aging and successful aging. Front Immunol.

[20] Lahita RG (2023). Sex and gender influence on immunity and autoimmunity. Front Immunol.

[21] Di Florio DN, Sin J, Coronado MJ, Atwal PS, Fairweather D (2020). Sex differences in inflammation, redox biology, mitochondria and autoimmunity. Redox Biol.

